# Supplementary material for: Indocyanine green fluorescence imaging-guided versus conventional laparoscopic lymphadenectomy for gastric cancer: long-term outcomes of a phase 3 randomised clinical trial
Source: Nat Commun. 2023 Nov 16;14:7413. doi: 10.1038/s41467-023-42712-6 (PMC10654517; doi:10.1038/s41467-023-42712-6)
Supplement: Supplementary file 2 — Reporting Summary [file 41467_2023_42712_MOESM2_ESM.pdf]

nature portfolio

Corresponding author(s): Chang-Ming Huang

Last updated by author(s): Oct 15, 2023

Reporting Summary

Nature Portfolio wishes to improve the reproducibility of the work that we publish. This form provides structure for consistency and transparency in reporting. For further information on Nature Portfolio policies, see our Editorial Policies and the Editorial Policy Checklist.

Statistics

For all statistical analyses, confirm that the following items are present in the figure legend, table legend, main text, or Methods section.

n/a	Confirmed	
☐	☒	The exact sample size (*n*) for each experimental group/condition, given as a discrete number and unit of measurement
☒	☐	A statement on whether measurements were taken from distinct samples or whether the same sample was measured repeatedly
☐	☒	The statistical test(s) used AND whether they are one- or two-sided<br>*Only common tests should be described solely by name; describe more complex techniques in the Methods section.*
☐	☒	A description of all covariates tested
☐	☒	A description of any assumptions or corrections, such as tests of normality and adjustment for multiple comparisons
☐	☒	A full description of the statistical parameters including central tendency (e.g. means) or other basic estimates (e.g. regression coefficient) AND variation (e.g. standard deviation) or associated estimates of uncertainty (e.g. confidence intervals)
☐	☒	For null hypothesis testing, the test statistic (e.g. *F*, *t*, *r*) with confidence intervals, effect sizes, degrees of freedom and *P* value noted<br>*Give P values as exact values whenever suitable.*
☒	☐	For Bayesian analysis, information on the choice of priors and Markov chain Monte Carlo settings
☐	☒	For hierarchical and complex designs, identification of the appropriate level for tests and full reporting of outcomes
☒	☐	Estimates of effect sizes (e.g. Cohen's *d*, Pearson's *r*), indicating how they were calculated

Our web collection on statistics for biologists contains articles on many of the points above.

Software and code

Policy information about availability of computer code

Data collection	All data were collected with Microsoft Excel.
Data analysis	All data were analyzed using SPSS statistical software, version 22.0 (SPSS Inc), and the R software environment, version 4.2.0 (R Foundation for Statistical Computing).

For manuscripts utilizing custom algorithms or software that are central to the research but not yet described in published literature, software must be made available to editors and reviewers. We strongly encourage code deposition in a community repository (e.g. GitHub). See the Nature Portfolio guidelines for submitting code & software for further information.

Data

Policy information about availability of data

All manuscripts must include a data availability statement. This statement should provide the following information, where applicable:

- Accession codes, unique identifiers, or web links for publicly available datasets
- A description of any restrictions on data availability
- For clinical datasets or third party data, please ensure that the statement adheres to our policy

The data supporting the findings in this study are available under controlled access due to data privacy laws related to patient consent for data sharing and the data should be used for research purposes only. All the original clinical data will be made available on request from the corresponding author (Huang CM) at any time in a de-identified manner. The remaining data are available within the Article, Supplementary Information. Request for data sharing will be handled in line with the

data access and sharing policy of Fujian Medical University Union Hospital. The original study protocol is available in the Supplementary Information as a Supplementary Note. Source data are provided with this paper.

Research involving human participants, their data, or biological material

Policy information about studies with human participants or human data. See also policy information about sex, gender (identity/presentation), and sexual orientation and race, ethnicity and racism.

Reporting on sex and gender	Sex and gender were not considered in the study design. Self-reported sex was collected and used in the analyses.
Reporting on race, ethnicity, or other socially relevant groupings	Race, ethnicity, or other socially relevant groupings were not considered in the study design.
Population characteristics	1.Age from 18 to 75 years<br>2.Primary gastric adenocarcinoma (papillary, tubular, mucinous, signet ring cell, or poorly differentiated) confirmed pathologically by endoscopic biopsy<br>3.Clinical stage tumor T1-4a (cT1-4a), N-/+, M0 at preoperative evaluation according to the American Joint Committee on Cancer (AJCC) Cancer Staging Manual Seventh Edition<br>4.No distant metastasis, no direct invasion of pancreas, spleen or other organs nearby in the preoperative examinations<br>5.Performance status of 0 or 1 on Eastern Cooperative Oncology Group scale (ECOG)<br>6.American Society of Anesthesiology score (ASA) class I, II, or III
Recruitment	Any gastric cancer patient who was admitted at the center, and met the criteria for inclusion was considered for recruitment, thus we confirm that there was no selection bias during recruitment. Patients were screened by the trained clinicians at the designated center and the principal investigators were responsible for the evaluation of pretreatment assessment and deciding for enrollment
Ethics oversight	This study was approved by the institutional review board of Fujian Medical University Union Hospital (IRB number: 2016YF015-02)

Note that full information on the approval of the study protocol must also be provided in the manuscript.

Field-specific reporting

Please select the one below that is the best fit for your research. If you are not sure, read the appropriate sections before making your selection.

☒ Life sciences ☐ Behavioural & social sciences ☐ Ecological, evolutionary & environmental sciences

For a reference copy of the document with all sections, see nature.com/documents/nr-reporting-summary-flat.pdf

Life sciences study design

All studies must disclose on these points even when the disclosure is negative.

Sample size	This study is a superiority test (unilateral), whose primary outcome measure is the total number of retrieving LNs. According to the previous study results and related literature reports, the total number of LN dissections in the control group was about 32.9, This analysis was based on an α of 0.05, a power of 80%, and a margin delta of 15%, revealing that at least 107 patients would be necessary per group. Considering an expected dropout rate of 20%, it was determined that each group needed at least 133 patients, for a total of 266 cases.
Data exclusions	1.Women during pregnancy or breast-feeding<br>2.Severe mental disorder<br>3.History of previous upper abdominal surgery (except laparoscopic cholecystectomy)<br>4.History of previous gastrectomy, endoscopic mucosal resection or endoscopic submucosal dissection<br>5.History of allergy to iodine agents<br>6.Enlarged or bulky regional LN diameter over 3cm by preoperative imaging<br>7.History of other malignant disease within past five years<br>8.History of previous neoadjuvant chemotherapy or radiotherapy<br>9.History of unstable angina or myocardial infarction within past six months<br>10.History of cerebrovascular accident within past six months<br>11.History of continuous systematic administration of corticosteroids within one month<br>12.Requirement of simultaneous surgery for other disease<br>13.Emergency surgery due to complication (bleeding, obstruction or perforation) caused by gastric cancer<br>14.FEV1[illegible]50% of predicted values<br>15.Linitis plastica, Widespread
Replication	All the described results were tested for replication by two independent statisticians.
Randomization	Eligible patients were randomly assigned by a 1:1 ratio to either the ICG or Non-ICG group. The data manager, who was not involved in the eligibility assessment and recruitment of patients, performed randomization with a list of randomly ordered treatment identifiers generated by a permuted block design using SAS (version 9.2; SAS Institute Inc.). The allocation sequence was concealed from the surgeons who enrolled

the patients until they were formally randomized to their groups. However, it was not feasible to blind the surgeons and participants owing to the nature of the surgical clinical trial.

Blinding: This research adopts an open design

Reporting for specific materials, systems and methods

We require information from authors about some types of materials, experimental systems and methods used in many studies. Here, indicate whether each material, system or method listed is relevant to your study. If you are not sure if a list item applies to your research, read the appropriate section before selecting a response.

Materials & experimental systems

n/a	Involved in the study
☒	☐ Antibodies
☒	☐ Eukaryotic cell lines
☒	☐ Palaeontology and archaeology
☒	☐ Animals and other organisms
☐	☒ Clinical data
☒	☐ Dual use research of concern
☒	☐ Plants

Methods

n/a	Involved in the study
☒	☐ ChIP-seq
☒	☐ Flow cytometry
☒	☐ MRI-based neuroimaging

Clinical data

Policy information about clinical studies

All manuscripts should comply with the ICMJE guidelines for publication of clinical research and a completed CONSORT checklist must be included with all submissions.

Clinical trial registration: ClinicalTrials.gov, NCT03050879

Study protocol: The protocol can be obtained from the article's supplement information

Data collection: All data were prospectively collected between November 19, 2018 to July 13, 2019 at Fujian Medical University Union Hospital, a tertiary referral teaching hospital for gastric cancer in Fujian, China

Outcomes:

Primary outcome
1.Total number of retrieved LNs
Secondary Outcome
1.The rate of fluorescence
2.Positive rate
3.False positive rate
4.Negative rate
5.False negative rate
6.Number of metastatic LNs
7.Metastatic rate of LN
8.Morbidity and mortality rates
9.3-year disease-free survival rate
10.3-year overall survival rate
11.3-year recurrence pattern
12.Postoperative recovery course
13.Operation time
14.The variation of weight
15.Intraoperative blood loss
16.Conversive rate
17.Intraoperative morbidity rates
18.Incision length
19.The variation of cholesterol
20.The variation of album
21.The results of endoscopy
22.The variation of body temperature
23.The variation of white blood cell count
24.The variation of hemoglobin
25.The variation of C-reactive protein
26.The variation of prealbumin
27.Recurrence pattern
