# Supplementary material for: Indocyanine green fluorescence imaging-guided versus conventional laparoscopic lymphadenectomy for gastric cancer: long-term outcomes of a phase 3 randomised clinical trial
Source: Nat Commun. 2023 Nov 16;14:7413. doi: 10.1038/s41467-023-42712-6 (PMC10654517; doi:10.1038/s41467-023-42712-6)
Supplement: Supplementary file 5 — Description of Additional Supplementary Files [file 41467_2023_42712_MOESM5_ESM.pdf]

Description of Additional Supplementary Files

Title: Supplementary Data 1

Description: Contains the clinical data of this study.
